# Electronic oral health surveillance system for Egyptian preschoolers using District Health Information System (DHIS2): design description and time motion study

**DOI:** 10.1186/s12903-024-04550-w

**Published:** 2024-07-16

**Authors:** Hams H. Abdelrahman, Maha Hamza, Wafaa Essam, May Adham, Abdulrahman AbdulKafi, Mohammad Baniode

**Affiliations:** 1 Department of Pediatric Dentistry and Dental Public Health, Faculty of Dentistry, Alexandria University, Champollion St., Azarita, 21526 Alexandria Egypt; 2 Health Information Systems Programme (HISP), Middle East and North Africa (MENA), Amman, Jordan; 3 Al Quds University, Jerusalem, Palestine

**Keywords:** DHIS2, Health systems, Oral Health Surveillance, Tele surveillance, Mobile Technology, m-Oral Health

## Abstract

**Background:**

Early childhood caries (ECC) is a major global health issue affecting millions of children. Mitigating this problem requires up-to-date information from reliable surveillance systems. This enables evidence-based decision-making to devise oral health policies. The World Health Organization (WHO) advocates the adoption of mobile technologies in oral disease surveillance because of their efficiency and ease of application. The study describes developing an electronic, oral health surveillance system (EOHSS) for preschoolers in Egypt, using the District Health Information System (DHIS2) open-source platform along with its Android App, and assesses its feasibility in data acquisition.

**Methods:**

The DHIS2 Server was configured for the DHIS2 Tracker Android Capture App to allow individual-level data entry. The EOHSS indicators were selected in line with the WHO Action Plan 2030. Two modalities for the EOHSS were developed based on clinical data capture: face-to-face and tele/asynchronous. Eight dentists in the pilot team collected 214 events using modality-specific electronic devices. The pilot’s team's feedback was obtained regarding the EOHSS's feasibility in collecting data, and a time-motion study was conducted to assess workflow over two weeks. Independent t-test and Statistical Process Control techniques were used for data analysis.

**Results:**

The pilot team reported positive feedback on the structure of the EOHSS. Workflow adaptations were made to prioritize surveillance tasks by collecting data from caregivers before acquiring clinical data from children to improve work efficiency. A shorter data capture time was required during face-to-face modality (4.2 ± 0.7 min) compared to telemodality (5.1 ± 0.9 min), *p* < 0.001). The acquisition of clinical data accounted for 16.9% and 21.1% of the time needed for both modalities, respectively. The time required by the face-to-face modality showed random variation, and the tele-modality tasks showed a reduced time trend to perform tasks.

**Conclusions:**

The DHIS2 provides a feasible solution for developing electronic, oral health surveillance systems. The one-minute difference in data capture time in telemodality compared to face-to-face indicates that despite being slightly more time-consuming, telemodality still shows promise for remote oral health assessments that is particularly valuable in areas with limited access to dental professionals, potentially expanding the reach of oral health screening programs.

**Supplementary Information:**

The online version contains supplementary material available at 10.1186/s12903-024-04550-w.

## Introduction

Oral diseases are a public health problem across the world as 3.5 billion people, representing 45% of the global population, suffer from oral diseases, with three out of four affected persons living in low- and middle-income countries [1]. Dental caries is the most significant contributor to the global burden of oral diseases. Early Childhood Caries (ECC) in primary teeth is the most prevalent chronic childhood disease [2]. It affects about 532 million infants, 48.1% in low-middle-income countries [2]. Untreated caries are associated with pain and inability to eat or sleep, which negatively impact the child’s daily activities, growth, self-esteem, and capacity to socialize [3].

Addressing ECC is a public health priority, necessitating current and reliable data through an effective monitoring mechanism [4]. This can be achieved by establishing public health surveillance systems that enable ongoing systematic collection, analysis, and interpretation of outcome-specific data for use in the planning, implementation, and evaluation of public health practice. Oral health surveillance systems empower the dental community to improve the oral health of the population in a manner that optimizes cost-effectiveness [5]. Data availability allows the identification of risk factors affecting diseases and, hence, developing or modifying oral healthcare programs and systems and setting priorities for allocating scarce health resources. Also, dental educators use data to develop educational programs and tailor them to community needs [6, 7]. 

There is a growing recognition of the pivotal role of digitalization in healthcare, particularly surveillance and data management [8]. The World Health Organization (WHO) advocates using mobile technologies to develop mobile Oral (m-Oral) Health surveillance programs and enable interoperable and coordinated data collection, offering feasible solutions for surveillance on a large scale [9]. Digital data collection not only demonstrates its feasibility but also is faster, often more reliable, user-friendly, and more economical than traditional data collection methods. Furthermore, it facilitates real-time data validation checks, enables remote data quality control, monitors field-based data collection progress, and facilitates immediate data analysis and access that enables better policy-making decisions [10]. 

The District Health Information System Version 2 (DHIS2) is one of these digital solutions. DHIS2 is a free, open-source, customizable platform that integrates seamlessly with mobile applications and has been deployed in 80 low and middle-income countries (LMICs) in routine health information systems and surveillance of epidemics [11]. DHIS2 empowers users to design data collection forms, add indicators, and customize data visualization tools while also offering dashboard functionalities to support aggregate and case-based disease surveillance capabilities [12]. Data regarding ECC are most scarce in LMICs [1], although these countries have the greatest experience of using the DHIS2 for surveillance. Thus, it may be possible to use the DHIS2 in oral disease surveillance by integrating oral health surveillance with existing surveillance systems for non-communicable diseases.

Implementing electronic surveillance systems necessitates training healthcare workers on the use of technology, data recording, and workflow [9]. Data recording remains an essential factor in the successful deployment of surveillance systems [13]. Shedding light on workflow patterns helps determine the time and workforce required for performing surveillance tasks [14]. Furthermore, assessing the time needed for documentation affects the feasibility of a surveillance system. This assessment can use resource management methodologies like time-motion studies (TMS) [15]. In a TMS investigation, an observer logs every action conducted by a healthcare professional, noting the duration of each action. This approach allows for accurate activity duration, frequency, timing, and sequence tracking, thus shedding light on the system's feasibility and providing insights about human resources requirements [14, 15]. 

In Egypt, the prevalence of ECC in children under five years old is more than 50% [16]. Information about ECC is sourced from published scientific research with no surveillance system assessing the distribution or trends of the disease [16]. This study aimed to describe the development of an electronic, oral health surveillance system (EOHSS) for preschool children in Egypt, using the DHIS2 with its Android mobile application. It also evaluates the feasibility of developing two electronic modalities for gathering clinical data and analyzing the time required for data acquisition. This study represents a pioneering effort, being the first attempt to use DHIS2 within the context of LMICs to establish an oral health electronic surveillance system.

## Materials and methods

### Setting and ethical approvals

This is a proof-of-concept study of the EOHSS prototype conducted in the pediatric dentistry clinics in the Faculty of Dentistry, Alexandria University, Egypt. According to the department statistics, these clinics provide free dental care to over 14,000 children annually, with a particular focus on underprivileged patients from at least two nearby governorates. The study proposal received ethical approval from the Research Ethics Committee at the Faculty of Dentistry, Alexandria University, Egypt. (#0724–7/2023). Informed consent was obtained from the parents before data collection.

### EOHSS development

#### Team and preparation phase

A piloting team of eight dentists, led by a team leader, was established to develop the EOHSS. The team leader contacted the Health Information System Project (HISP) Centre at the University of Oslo, Norway, to ensure support from the HISP Middle East and North Africa (MENA) office. Six online meetings over six months, from August 2023 to January 2024, were conducted for needs assessment to develop the EOHSS. The meetings identified the objectives, elements, and indicators of the EOHSS, the electronic devices to be used, and the requirements of the hosting servers. The team also brainstormed about mobile data collection and the configuration of electronic forms.

#### Design of the EOHSS

The DHIS2 Tracker Capture application was used to allow individual-level data entry. This open-source tool could be easily set up on Android tablets or smartphones, ensuring flexibility in data collection at service delivery points. The DHIS2 Server was configured for the DHIS2 Android Capture App [17]. DHIS2 is packaged as a standard Java Web Archive file. It runs on any Servlet container where Java Runtime Environment version 8 or higher is installed, providing a web server and a database server with sufficient memory and storage space. DHIS2 is a liberal BSD 3-clause license and is available from https://dhis2.org/downloads/. We used the recommended software ecosystem for the production server, the Ubuntu 16.04 LTS operating system, the PostgreSQL database, and the Tomcat Servlet. We also used the DHSI2 Android tracker version 2.9, which could be downloaded from https://github.com/dhis2/dhis2-android-capture-app/releases or https://play.google.com/store/apps/details?id=com.dhis2&hl=en_US.

### The EOHSS had the following features:

#### Data Elements

We used the following sources to identify indicators for the EOHSS: WHO Global Oral Health Action Plan 2030 [18], the WHO Oral Health Surveys Manual [19], and the Association of State and Territorial Dental Director (ASTDD) Basic Screening Survey for Children [20]. The indicators assessed the oral health of preschool children in various categories, including (1) oral health outcomes, (2) dental treatment needs, (3) access to care, (4) oral health risk factors, and (5) oral health-related quality of life. Indicators were selected by a panel of five specialists in dental public health and pediatric dentistry. The panel rated the indicators regarding alignment with the predefined areas and the importance of monitoring the oral health of preschool children. Thirteen indicators out of 30 potential indicators were eventually selected. (Supplemental File 1).

#### Data acquisition modalities

**Fig. 1:**
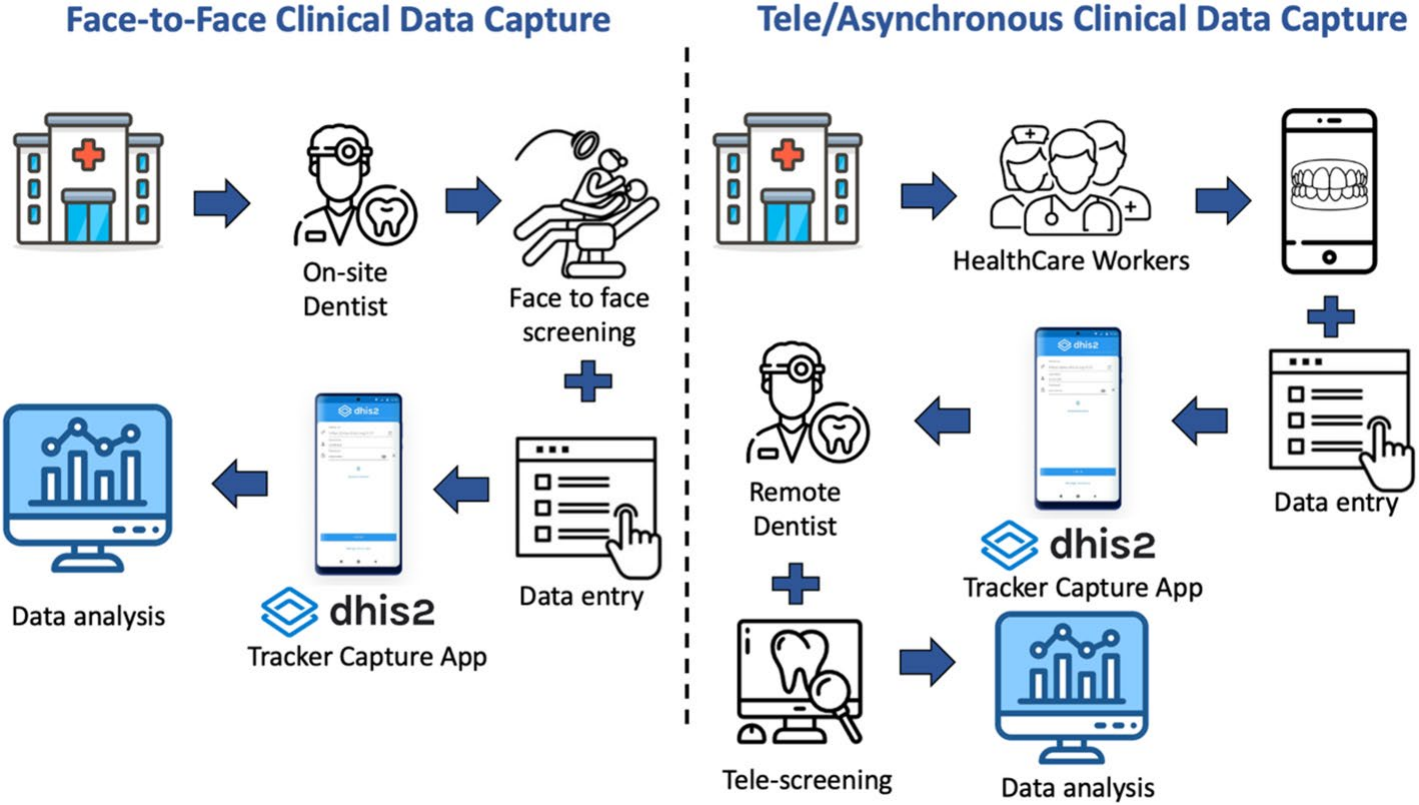
Two Modalities of the EOHSS

Two modalities for clinical data capture were developed (Fig. 1).

- Face-to-face examination
  In this modality, dental professionals collect clinical data using face-to-face examinations of children. The dentists input text or numerical data of the indicators into the DHIS2 Tracker Capture application utilizing Android tablets or smartphones.

b) Tele/Asynchronous (store and forward) examination
  This modality was developed to reduce the need for dentists to collect clinical data. A non-dentist (e.g., assistant, nurse, etc.) captures intra-oral photographs of the child’s mouth using the DHIS2 Tracker application via smartphones only. The child’s data are stored on the hosting server and retrieved by remote dentists to assess the clinical condition and enter the indicators’ text or numerical data.

#### Devices and connectivity

The DHIS2 generic specifications for Android tablets and smartphones used for data acquisition are: should run on at least Android 7, have four cores – a 1.2 GHz processor, a RAM capacity of 2 GB, a storage space of 32 GB, and at least 8Mpx Camera with 4G (LTE) [21]. We used higher specifications to support photographing the intraoral structures: triple rear cameras, a 48 MP wide primary lens, an 8 MP ultrawide-angle lens, and a 2 MP depth lens. Data acquisition proceeded offline and was later synched to the database. Additionally, a " share " functionality was embedded in the sign-in interface to generate a QR code. When another device scans this QR code, data can be transferred to another secure device to continue data entry if the first device malfunctions for any reason.

#### Organizational Units

In DHIS2, the spatial dimension of data reflecting the geographical context is represented by organizational units. The EOHSS will be deployed in one governorate, Alexandria, and therefore, a restricted set of organizational units was established, encompassing only one hierarchical level, the governorate level. The EOHSS was divided into units, and each unit represented a different public healthcare facility where data would be collected.

#### Database accessibility, security, and quality control

Several security features were implemented, including (1) password protection and encryption of backups for data collection, (2) use of robust usernames and passwords for user authentication, thereby safeguarding the confidentiality of data, even in the event of device loss, (3) daily synchronization of data with local servers via secure connections and (4) prohibition of screenshot capture or screen sharing to maintain patient privacy [22]. 

The DHIS2 offers access rights for different user roles [23]. Distinct access levels were developed to allow users with different roles to access different features in the two data acquisition modalities. In the face-to-face EOHSS, there were two levels of access: level 1 for the dentist to enable data entry for screened children, and level 2 for the upper management with access to individual data entered by the dentist, aggregated summaries across all organizational units, inclusive of DHIS2 data visualization capabilities and reporting. In the tele/asynchronous EOHSS, there were three access levels. Level 1 for healthcare workers for non-clinical data entry and capturing intra-oral photographs, level 2 for dentists to remotely assess the clinical oral health status based on photographs, and level 3 mirrored the access privileges of level 2 in the face-to-face EOHSS.

Validation controls were incorporated into the EOHSS to ensure data quality. These were based on data type and value range (such as the number of teeth) and marked mandatory data fields with error messages.

#### The EOHSS Screens

**Fig. 2:**
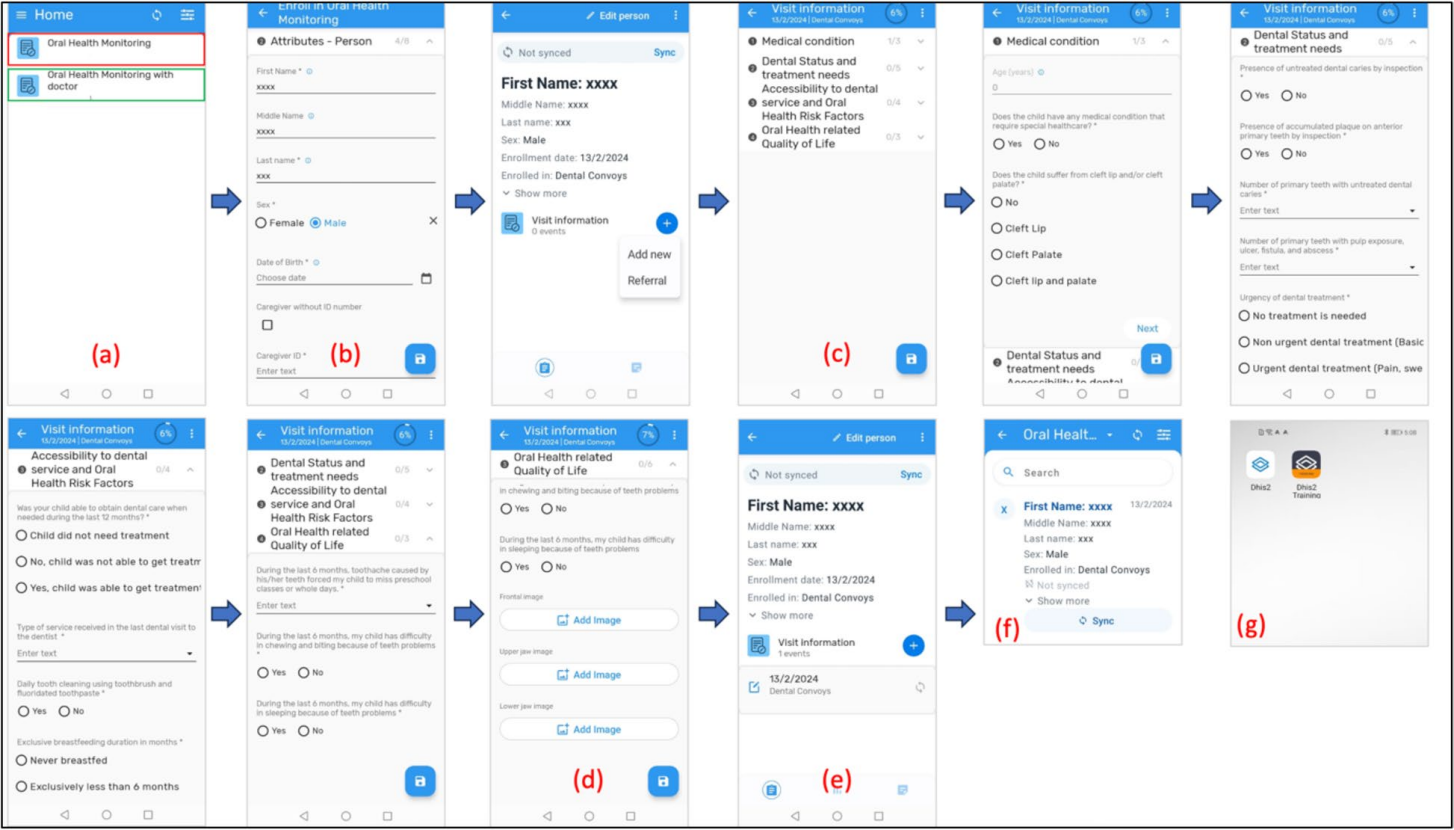
EOHSS simulation using a training iteration—no patient data included (a) Home page including both surveillance modalities (Red frame includes Face-to-Face EOHSS, Green frame includes Tele/Asynchronous EOHSS), (b) Registration form with child demographic data and organizational unit, (c) Main form with drop-down sections containing all oral health indicators, (d) Fields for the intraoral photographs in the tele/ asynchronous modality, (e) Completed form, (f) List showing a submitted record that requires synchronization, (g) DHIS2 Android App Icons

**Fig. 3:**
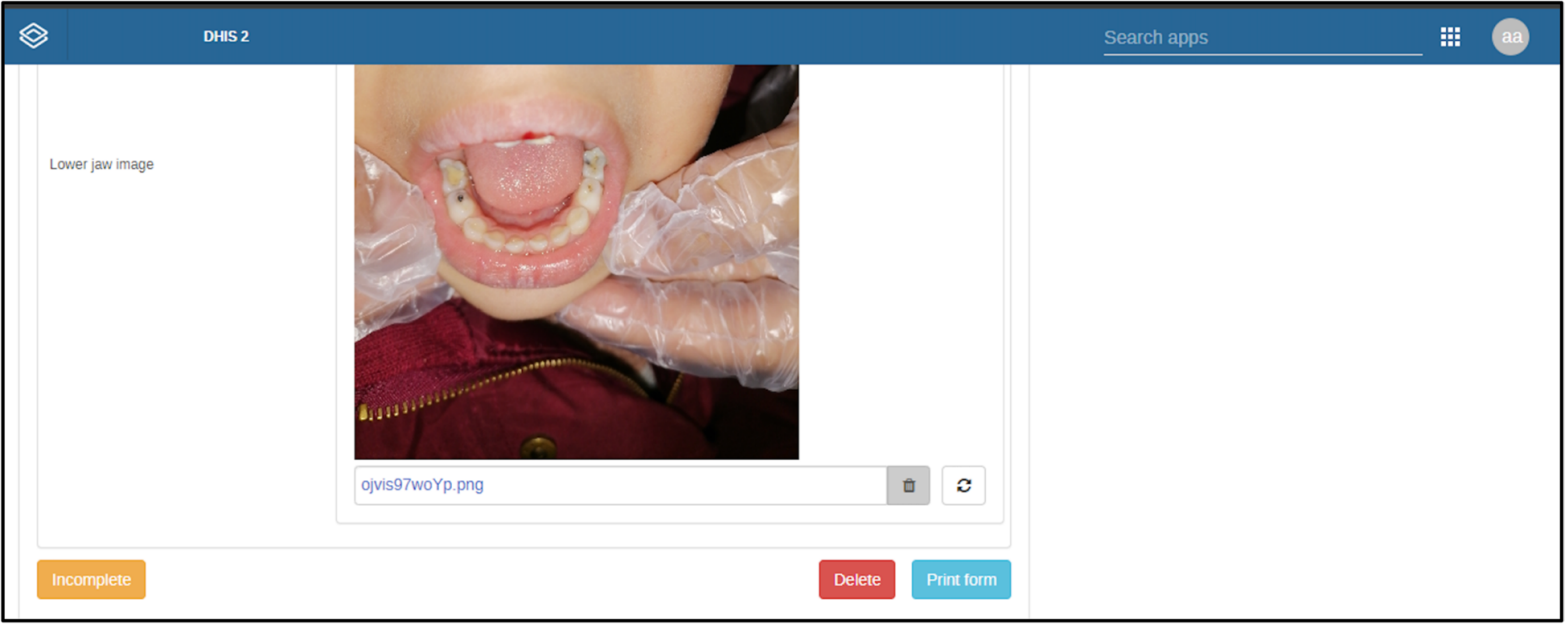
Example of an intraoral photograph to be assessed by a remote dentist

**Fig. 4:**
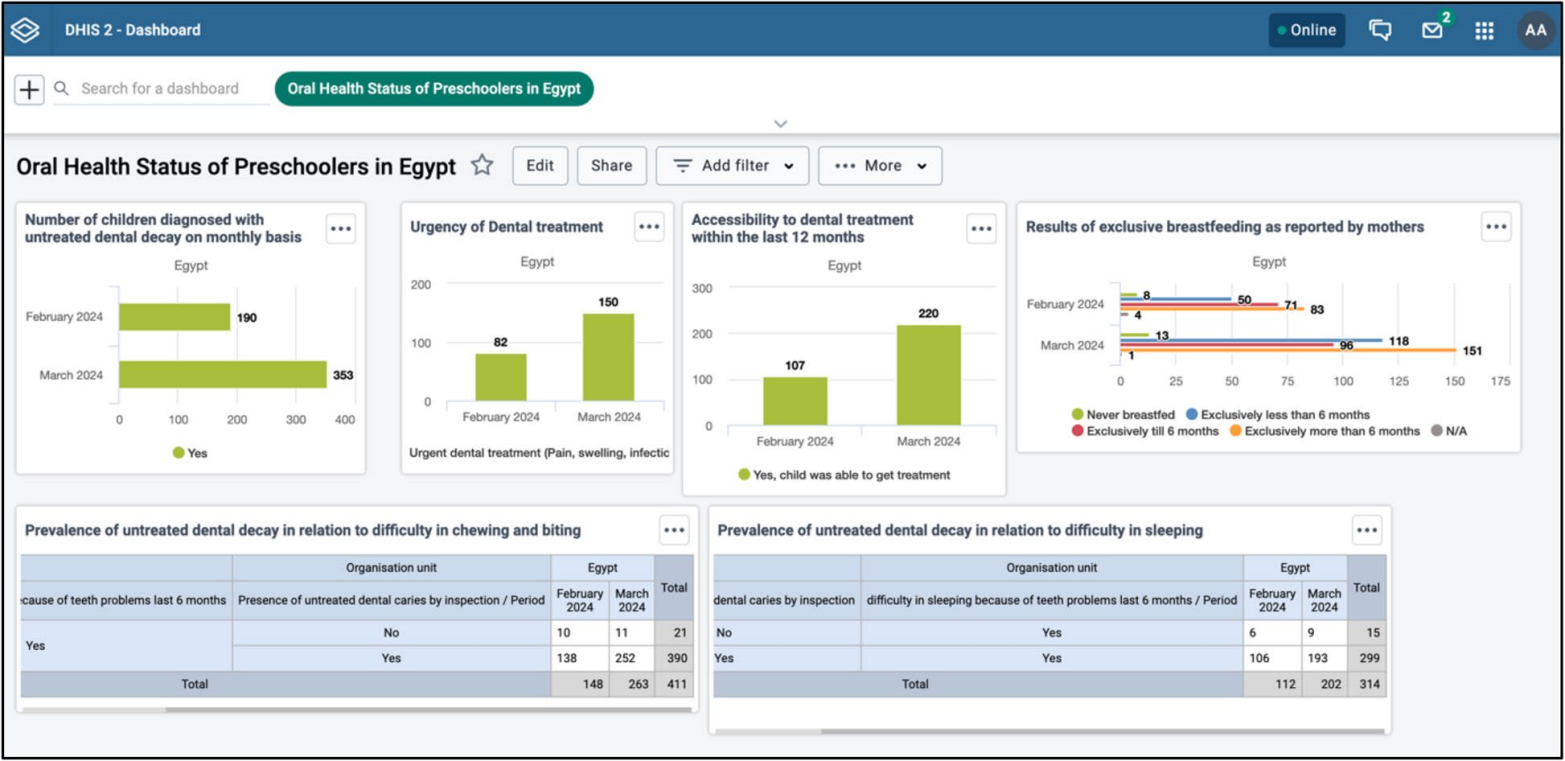
Example of the dashboard showing a report with different data elements

The EOHSS is included in two electronic pages. (Fig. 2) The first page consists of child identifiers, including the child's age, sex, birth date, caregiver’s ID, and caregiver’s contact number (Fig. 2a). The second page consists of five categories in a single page with drop-down sections (Figs. 2c-e):

- Medical history, recording information about systemic conditions, and cleft lip and cleft palate.
- Dental status, treatment needs, and clinical data must be obtained from the face-to-face examination and dentists’ assessment of the oral photographs. (e.g., presence of caries and pufa index)
- Dental visits and oral health risk factors record information about the accessibility of dental care, fluoride exposure, and breastfeeding practices.
- Oral Health-Related Quality of Life based on three questions to evaluate the impact of oral health on the child’s quality of life.
- In the tele/asynchronous modality, a fifth category was added to accommodate three intraoral photographs (frontal view, upper jaw, and lower jaw photos). A remote dentist uses a web-based interface to review the uploaded photographs and fill in the dental assessment section. (Fig. 3)
- Dashboard: The system allows instantaneous data analysis for validated records using visualization tools in the dashboard, which supports tabular and graphical presentations. This dashboard can be built for each organization unit separately. The dynamic dashboards empower users to visualize data effectively for comprehension and communication with stakeholders. They allow users to generate reports based on the data elements and indicators that the oral health surveillance program has predefined. Users can apply filters to metadata, selecting specific elements, indicators, time frames, etc., to tailor the visualization to their needs. (Fig. 4)

### Data acquisition

The piloting team trained on using the EOHSS under the supervision of the team leader over five days. The team was provided with theoretical instruction via online sessions explaining the interface and the two modalities, followed by a demonstration and onsite training in the pediatric dental clinics. The team underwent calibration training on 20 children using standardized clinical data collection criteria to ensure reliability in dental status assessment [24]. Weighted kappa scores ranged from 0.701 to 0.846, indicating substantial intra-examiner agreement.

Over two weeks, 214 surveillance events were recorded, on an average of 5 h daily. We included a convenience sample of 214 children equally allocated to the two modalities. Children were included if they were older than one year and younger than six years of both sexes with no other exclusion criteria.

Four tablets and three smartphones were used with different usernames and passwords. Upon successful authentication, users had access to the electronic forms. Data were collected offline, and synchronization was performed at the end of each day after data collection. The team leader reviewed all records to monitor data entry progress and quality on different days.

### EOHSS assessment and analysis of findings

The piloting team participated in the preliminary testing of the two surveillance modalities. This team has already become familiar with the EOHSS during training for data acquisition. Feedback was gathered from the piloting team about the architecture of data forms, the feasibility of clinical data capture using the two modalities, and the workflow efficiency appropriate for needs assessment and design modification [25]. A time-motion study assessed workflow using the EOHSS over two weeks from 21st January to 7.^th^ February 2024. To train for time assessment, the team leader timed the EOHSS tasks for ten children and compared her timing to two different team members. The intraclass correlation coefficient between them was ≥ 0.92. No intra-observer agreement in timing was conducted because the EOHSS prevented duplicate data entry. The team leader recorded the duration of each task using a stopwatch and entered this into a sheet containing all tasks for each child. The observer shadowed the pilot team members for 5 h daily, each day of the two weeks over the data acquisition period. Data were entered into an Excel sheet by the end of each day. Motion assessment involves identifying the tasks and their naturally adopted sequence to complete the process. The tasks used to acquire data for the EOHSS were classified following a sequential three-step process: initial field observation by the team leader during the training period to identify contextually relevant activities, subsequent validation through repetitive execution of tasks over two days using the two modalities and final application of the validated classification system during testing [26]. 

To analyze the time-motion study findings, the normality of time in minutes was assessed using the Kolmogorov–Smirnov test and Q-Q plots. Normal distribution was confirmed, and data were presented using mean and standard deviation (SD). The two surveillance modalities were compared regarding total time and time per task using independent t-test. The proportion of time spent on each task was calculated out of the total time. Consecutive children examined in the study were considered a proxy of progress in gaining experience with the EOHSS. Variation of the total time across consecutive children was assessed using Statistical Process Control (SPC) techniques. SPC uses charts that show the departure from normal variation when the time goes beyond control limits. These limits were set at (1σ), (2σ), and (3σ), corresponding to 1, 2, and 3 standard deviations away from the mean and 8 consecutive points below the center line, indicating the average was also identified. All tests were two-tailed, and the significance level was set at *p*-value < 0.05. Data were analyzed using IBM SPSS for Windows, version 23, Armonk, NY, USA.

## Results

The feedback obtained from the piloting team revealed overall agreement about the simplicity of the data entry process utilizing both modalities, the structured layout of electronic forms, and the user-friendly interface of tablets and smartphones. The tasks used in the EOHSS were observed and classified as described in Table 1.

**Table 1:** Surveillance tasks observed during the time motion study

	Description of tasks	
	Face-to-face EOHSS	Tele/asynchronous EOHSS
Verbal Communication	Communication with caregivers for registration and acquisition of non-clinical data	
Direct patient contact	Face-to-face examination by dentist	Capturing photographs of intra-oral structures: upper arch, lower arch and frontal view
Health information management	-	Clinical photographs screening by remote dentist
	Transition from child registration section to main form	
	Submission of the completed form	
Infection control	Changing gloves and washing hands with hand sanitizer	

Through observation, it was noted that the piloting team first discussed the sequence of tasks and decided to adhere to the order outlined in the electronic form. However, they reported less efficiency during the face-to-face EOHSS data entry when a dental examination was conducted before the caregiver’s interview because it took longer. Consequently, the team revised the sequence of tasks so that child registration, medical history, and caregiver interviewing preceded the face-to-face dental examination or intraoral photo capture. This modification reduced distractions and enhanced operational efficiency. Additionally, within face-to-face EOHSS data capture, dentists operated in pairs, one entering child registration and caregiver interview data and the other conducting the clinical oral examination. During the tele EOHSS data capture, one dentist managed the whole process.

Table 2 shows that using this workflow, the overall time for data capture in the face-to-face modality was shorter than the tele modality (mean 4.21 and 5.07 min, respectively, p < 0.001). The onsite tasks took about the same time, with the face-to-face modality taking a shorter time than the telemodality (mean difference 0.23 min).

**Table 2:** Time in minutes per task and proportion in relation to the overall time spent performing EOHSS tasks

**Categories**	**Tasks**	**Time in minutes** Mean ± SD		**F2F-Tele**	***p*** **value**	**Proportion of time %**	
		**F2F EOHSS**	**Tele EOHSS**			**F2F EOHSS**	**Tele EOHSS**
Verbal communication with caregivers	Registration	1.47 ± 0.40	1.42 ± 0.37	0.05	0.34	34.9%	28.0%
	Medical History	0.22 ± 0.11	0.20 ± 0.11	0.02	0.32	5.2%	3.9%
	Acquisition of non-clinical data	1.10 ± 0.31	1.05 ± 0.36	0.05	0.26	26.1%	20.7%
Direct patient contact: Dental examination or intraoral photographing		0.71 ± 0.32	1.07 ± 0.34	-0.36	< 0.001*	16.9%	21.1%
Health information management	Assessment of photographs by remote dentist	-	0.63 ± 0.28	-	-	-	12.4%
	Transition from child registration section to main form	0.09 ± 0.03	0.09 ± 0.03	-	-	2.1%	1.8%
	Submission of completed form	0.08 ± 0.02	0.08 ± 0.02	-	-	1.9%	1.6%
Infection Control		0.53 ± 0.22	0.53 ± 0.22	-	-	12.6%	10.5%
Total time	Onsite tasks only^a^	3.68 ± 0.74	3.91 ± 0.78	-0.23	0.04*	-	-
	All tasks	4.21 ± 0.74	5.07 ± 0.85	-0.86	< 0.001*	-	-

^*^Statistically significant difference at p value < 0.05, *F2F* face to face, ^a^Time is calculated without considering infection control and not including the assessment of intraoral photographs by a remote dentist

Registration took the longest time (about 1.4 min) in both modalities, constituting one-third of the time in both modalities. Also, the acquisition of non-clinical data accounted for 26.1% and 20.7% of the face-to-face and tele modalities.

The most significant difference between both modalities was in direct patient contact tasks, which was 0.36 min shorter in the face-to-face than in the tele modality. This time represented 16.9% and 21.1% of the time in both modalities, respectively. When the time to assess the photographs by the remote dentist was added, the difference in time between the face-to-face and the tele modalities in clinical data acquisition was 0.99 min. Infection control took 0.53 min, representing 10.5%- 12.6% of the time in both modalities.

Table 3 illustrates changes in task times over two weeks of pilot testing. Among tasks of the face-to-face modality, the time for taking medical history and acquiring non-clinical data was significantly reduced in the second week, and the registration and dental examination time demonstrated a slight non-significant increase (mean 0.07 and 0.11 min, respectively). In contrast, the telemodality showed a significant decrease in all tasks except registration, where the reduction was insignificant. The acquisition of non-clinical data and the assessment of intraoral photographs showed the most significant reduction (mean 0.30 and 0.24 min, respectively).

**Table 3:** Change in time in minutes per EOHSS task over two weeks of pilot testing

	**Tasks**		**1st week**	**2nd week**	**Difference**	***p* value**
**Face to face modality**	Number of children	n (%)	53 (49.5%)	54 (50.5%)	-	-
	Registration	Mean ± SD	1.44 ± 0.39	1.51 ± 0.41	0.07 ↑	0.34
	Medical History		0.27 ± 0.12	0.16 ± 0.08	0.11 ↓	< 0.0001*
	Acquisition of non-clinical data		1.17 ± 0.34	1.04 ± 0.26	0.13 ↓	0.03
	Dental Examination		0.66 ± 0.31	0.76 ± 0.29	0.11 ↑	0.07
	Total time		3.54 ± 0.79	3.47 ± 0.64	0.06 ↓	0.66
**Tele/Asynchronous modality**	Number of children	n (%)	61 (57%)	46 (43%)	-	-
	Registration	Mean ± SD	1.47 ± 0.40	1.36 ± 0.32	0.12 ↓	0.11
	Medical History		0.25 ± 0.11	0.15 ± 0.07	0.10 ↓	< 0.0001*
	Acquisition of non-clinical data		1.18 ± 0.35	0.88 ± 0.30	0.30 ↓	< 0.0001*
	Taking intraoral photograph		1.13 ± 0.39	1.00 ± 0.26	0.13 ↓	0.049
	Assessment of intraoral photograph		0.73 ± 0.32	0.49 ± 0.14	0.24 ↓	< 0.0001*
	Total time		4.76 ± 0.90	3.87 ± 0.63	0.89 ↓	< 0.0001*

^*^Statistically significant difference at *p* value < 0.05, **↑** increase in time, **↓** decrease in time

**Fig. 5:**
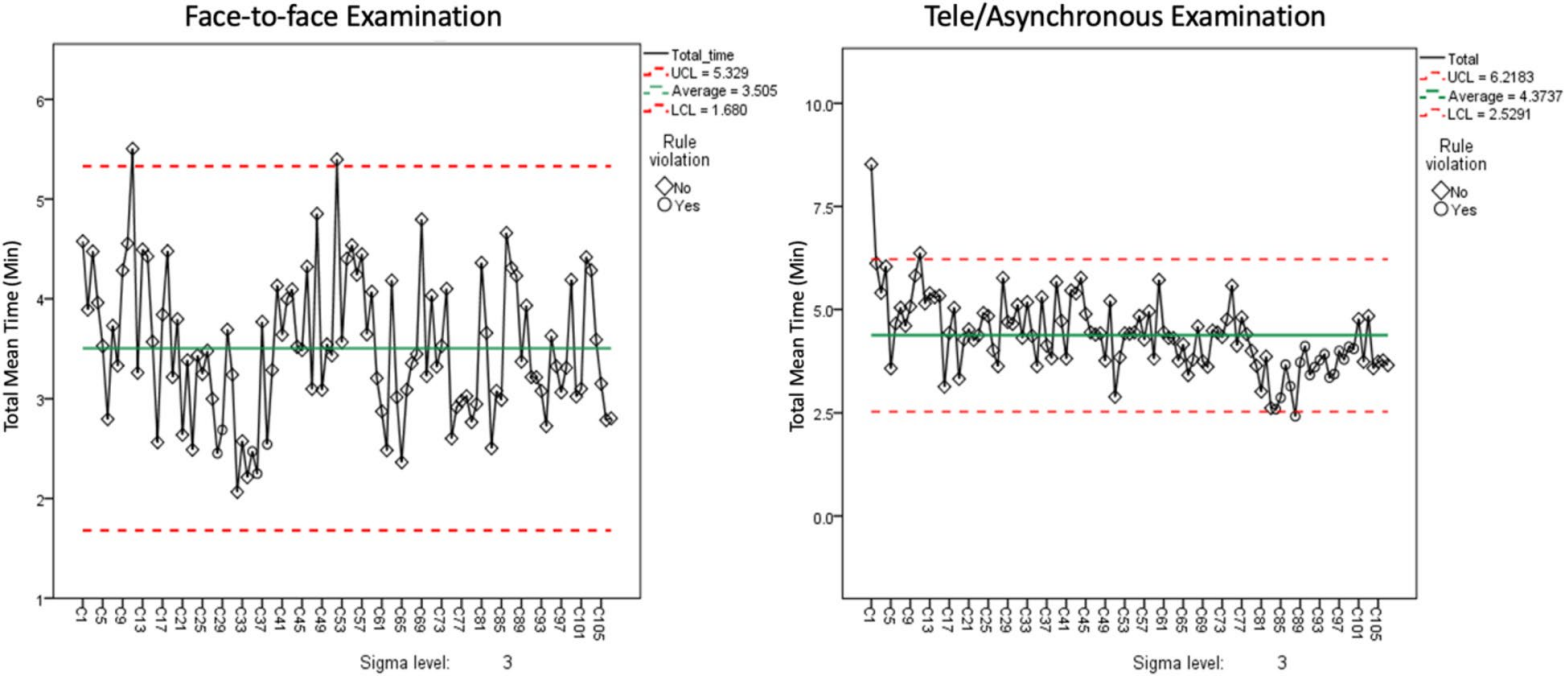
Comparison of the variation in time to perform tasks of the Face-to-Face (Left) and tele modalities (Right)

Figure 5 shows the variation in the overall time to perform the EOHSS tasks over two weeks to enter the data of 107 children. The time for the face-to-face modality showed random variation with no increase or decrease patterns. In the telemodality, starting from child #83, there was a noticeable decrease in time below the 2-sigma level. This reduction continued, fluctuating between 1-sigma and 2-sigma levels until child # 91, where eight consecutive time points were below the average, with a pattern suggestive of reduction in the second week.

## Discussion

Reducing ECC is a public health priority, especially in Egypt, and requires reliable and efficient surveillance systems to set appropriate health policies and strategies at the national and community levels [16, 18]. The current findings showed that the piloting team reported positive feedback regarding the EOHSS structure and the ease of using the two modalities and modified the workflow to perform tasks with the caregiver before acquiring clinical data from the child. One minute less was needed with the face-to-face modality than with the telemodality, and most of the time was spent verbally communicating with the caregiver. The time required for the face-to-face modality showed random variation across time, and the tele-modality tasks showed a trend of reduced time to perform tasks, especially in the acquisition of non-clinical data and assessment of intraoral photographs. This proof-of-concept study demonstrated the feasibility of using the DHIS2 to support an EOHSS for preschool children and the possibility of developing an efficient workflow using different clinical data capture modalities to complete a surveillance event in a short period.

Managing the flow of tasks can impact the quality of gathered data; therefore, in the current research, establishing an effective workflow was essential to improve productivity and data accuracy and limit the burden on the surveillance team [27]. Implementing a well-defined workflow can facilitate the seamless integration of electronic systems into organizational processes, fostering a culture of technology adoption among healthcare providers [28]. Therefore, carefully defining, refining, and training on workflow is essential to the success of EOHSS deployment. Capitalizing on the critical need for rapid data entry in health surveillance systems [5], the study achieved sub-6-min completion times for both EOHSS modalities. Tasks involving persons other than the child and healthcare provider, like child registration and caregiver interviews, took more time because they were only partially under the control of the healthcare personnel. These tasks ask caregivers to recall and disclose health information and face uncertainties regarding using shared data [29]. On the other hand, tasks handled solely by healthcare providers, like the remote assessment of photographs, took less time and improved further as dentists gained experience. Tasks shared by the healthcare providers and the children, like dental examinations and intraoral photography, occupied a middle ground because the child's cooperation influenced task duration. However, their time also decreased as proficiency increased over time, and the task became less intimidating to children. This highlights the importance of thorough training in the various tasks [9]. 

There is limited evidence on using mobile technology to support oral health surveillance. The existing electronic systems mainly rely on electronic dental records (EDR) or mobile applications adapted for surveillance objectives [5, 30, 31]. Although these systems provide real-time data, their utility is limited by several factors, including unstructured data and diverse semantic structures, data accessibility and confidentiality issues, incompatibility with other systems, inability to provide district-level estimates, and their reliance on proprietary software with vendor lock-ins, and limited potential as health surveillance systems [32]. By contrast, DHIS2 is a free, open-source global public good accessible without license restrictions. It can be adapted to local context and use cases, eliminating the necessity for core programming adjustments [33]. It is, thus, appropriate for developing surveillance systems in LMICs where investment in health information is limited [34]. Numerous studies have documented the utilization of DHIS2 as a disease surveillance system in LMICs at both national and subnational scales [12, 35–37]. However, no previous reports of its use to support oral health surveillance could be identified.

Incorporating photographs in this study, facilitated by the DHIS2’s capability to receive images, paves the way for a tele-surveillance system. The development of this system enables dentists to assess the oral health status of children remotely, thus overcoming geographic barriers, particularly in regions with dentist shortages, enhancing surveillance scalability, and potentially reducing workforce costs since non-dentists may capture clinical and non-clinical data [38]. While traditional field screening often focuses on fundamental dental indicators due to time constraints, a tele-surveillance system would offer the potential to assess a broader range of dental conditions, including caries at various stages, fluorosis, types of restorations, malocclusion, and gingival inflammation, thus, the remote assessment of these conditions may be more time-efficient and could enable the development of a comprehensive surveillance system and allow data to be used for clinical research [30]. 

Moreover, the study highlights the critical role of selecting the appropriate indicators to capture the targeted condition, thereby avoiding information overload at high organizational levels [39]. The indicators utilized in the EOHSS were chosen in alignment with global recommendations. However, they needed modification when used in the tele EOHSS. For instance, the *pufa* index necessitated the retraction of oral structures to allow examination of the oral vestibule. This increased the number of intraoral photographs needed to capture clinical data and required intraoral mirrors and mouth retractors, with additional cost and time [40]. To address this challenge, the examination may focus on teeth with high caries susceptibility and use low-cost equipment such as tongue retractors, provided that no essential information is lost to allow comprehensive assessment. Another factor to be considered is the impact of the smartphone camera and the intensity of lighting from the camera flash that could affect the clarity of images captured for the remote assessment [41]. This investigation underscores the criticality of selecting appropriate device specifications aligned with the intended surveillance objectives. Furthermore, it emphasizes the importance of factoring in the healthcare provider’s user experience to ensure efficient capturing of high-quality images that adhere to pre-defined criteria.

The study has some limitations. First, it was designed as a proof of concept with limited possibility to include patients from diverse backgrounds, and data were collected from a single institution to determine general design considerations, yet it is still being determined whether our results will be transferable to other institutions. Consequently, the extrapolation of findings beyond this specific context is not advised. Second, the study included a relatively small sample size of children and dentists. However, this was considered acceptable for studies on technology design as the goal was to pragmatically sample to obtain sufficient information for the iterative design process [25]. Third, our study focused on a specific pilot team; therefore, future studies must involve dental healthcare providers who will eventually use the EOHSS to assess their preparedness to integrate the system into their current surveillance activities. Fourth, the presence of an external observer during surveillance tasks may have influenced participants to alter their performance. While this method is optimal for non-centralized short tasks that yield detailed field data, it can be conducted asynchronously, with the observer analyzing video recordings of subject behavior [15]. 

Another limitation of its technical nature is that the DHIS2 tracker app is compatible only with Android operating systems, which restricts its use on iOS devices. Therefore, developing an iOS-compatible version would significantly promote accessibility, usability, and integration of the DHIS2 system in diverse healthcare environments and align with the evolving technological landscape and user preferences. In addition, the duration of the completion of the tasks in the tele EOHSS is affected by the number of photographs, and this, in turn, impacts the accuracy of reporting on the clinical indicators. Further studies are recommended to identify the number and positioning of the photographs needed to maximize the accuracy of disease detection compared to face-to-face examination. Finally, the study could not adequately assess the appropriateness of the dashboard because it was accessible to only one user, the team leader. Future assessment of this aspect of the EOHSS is needed.

## Conclusions

This study demonstrated the feasibility of an electronic surveillance system that could be accessed using different electronic devices to monitor the oral health of preschool children. The short time needed to use the system and the flexible workflow supported by technology, which reduces the need for dentists in the field, enable the establishment of an EOHSS that ensures the availability of reliable and timely oral health data.

## Future directions

Building on the successful customization and pilot testing of the DHIS2 tracker system for EOHSS, studies are required to estimate the net financial benefits of the two surveillance modalities and to assess the factors influencing the system's implementation and its adoption by healthcare workers. The focus will be on expanding the system to additional healthcare facilities and integrating it with national health information systems. Continuous training and capacity building for healthcare professionals, along with routine evaluation and system updates, to maintain data quality and effectiveness. Utilizing research data to inform policy, enhance community engagement, and secure long-term funding to ensure sustainability.

### Supplementary Information

Supplementary Material 1.

## Data Availability

The datasets used and /or analyzed during the current study are available from the corresponding author upon reasonable request.

## Abbreviations

ECC: Early Childhood Caries
DHIS2: District Health Information System – Version 2
EOHSS: Electronic Oral Health Surveillance System
LMICs: Low Middle-Income Countries
WHO: World Health Organization
TMS: Time Motion Study
ASTDD: Association of State and Territorial Dental Director
SPC: Statistical Process Control
EDR: Electronic Dental Records
