# Supplementary material for: Electronic oral health surveillance system for Egyptian preschoolers using District Health Information System (DHIS2): design description and time motion study
Source: BMC Oral Health. 2024 Jul 16;24:807. doi: 10.1186/s12903-024-04550-w (PMC11253332; doi:10.1186/s12903-024-04550-w)
Supplement: Supplementary file 1 — Supplementary Material 1. [file 12903_2024_4550_MOESM1_ESM.docx]

**Full Description of Oral Health Indicators for Preschool Children**

**“Tracker system”**

|  | Preschool Children | |
| --- | --- | --- |
|  | Birth | Head start |
| Sociodemographic Data | - First name: Middle Name: Last name: - ID caregiver No - Phone number - Child’s BirthDate - Sex: Male/Female | |
| Medical Condition | Does the child have any medical condition that require special healthcare? (Systemic disease, mental disability, ..etc)   - Yes/No | |
|  | Does the child suffer from cleft lip and/or cleft palate?   - None - Cleft lip - Cleft palate - Cleft lip and palate | |
| Dental status and treatment needs | Presence of untreated dental caries by inspection   - Yes/No   Presence of accumulated plaque on anterior primary teeth by inspection   - Yes/No   Number of primary teeth with untreated dental caries   - From 0 to 20   Number of primary teeth with pulp exposure, ulcer, fistula, and abscess   - From 0 to 20   Urgency of dental treatment   - No treatment is needed - Non-urgent treatment - Urgent treatment - General Anaesthesia | |
| Dental Visits and Oral Health Risk Factors | Was your child able to obtain dental care when needed during the last 12 months?   - The child did not need treatment - No, the child was not able to get treatment - Yes, the child was able to get treatment   Type of service received in the last dental visit to the dentist   - The child did not visit the dentist - No service received - Diagnosis and medication - Preventive treatment - Restorative treatment - Extraction - Appliance   Daily teeth cleaning using a toothbrush and fluoridated toothpaste   - Yes/No   Exclusive breastfeeding duration in months   - Child never breastfed - Less than 6 month - Till 6 months - More than 6 months | |
| Oral Health related Quality of Life | During the last 6 months, toothache caused by his/her teeth forced my child to miss preschool classes or whole days.   - Yes/No   During the last 6 months, my child has had difficulty in chewing and biting because of teeth problems   - Yes/No   During the last 6 months, my child has difficulty in sleeping because of teeth problems   - Yes/No | |

The pufa index scores the presence of a visible pulp (p), ulceration of the oral mucosa due to root fragments (u), a fistula (f), or an abscess (a)
